# Possible Use of Body Surface Area Value for Estimating Skeletal Muscle Mass in Chronic Liver Disease

**DOI:** 10.3390/diagnostics15030263

**Published:** 2025-01-23

**Authors:** Kazunori Yoh, Takashi Nishimura, Naoto Ikeda, Tomoyuki Takashima, Nobuhiro Aizawa, Yukihisa Yuri, Taro Kimura, Kohei Yoshihara, Ryota Yoshioka, Shoki Kawata, Yuta Kawase, Ryota Nakano, Hideyuki Shiomi, Shinya Fukunishi, Shinichiro Shinzaki, Shuhei Nishiguchi, Hirayuki Enomoto

**Affiliations:** 1 Department of Gastroenterology, Hyogo Medical University, Mukogawa-cho 1-1, Nishinomiya 663-8501, Japan; mm2wintwin@yahoo.co.jp (K.Y.); tk-nishimura@hyo-med.ac.jp (T.N.); nikeneko@hyo-med.ac.jp (N.I.); tomo0204@hyo-med.ac.jp (T.T.); aizawa-n@hyo-med.ac.jp (N.A.); yu-yukihisa@hyo-med.ac.jp (Y.Y.); ta-kimura@hyo-med.ac.jp (T.K.); ko-yoshihara@hyo-med.ac.jp (K.Y.); ri-yoshioka@hyo-med.ac.jp (R.Y.); sh-kawata@hyo-med.ac.jp (S.K.); yt-kawase@hyo-med.ac.jp (Y.K.); ri-nakano@hyo-med.ac.jp (R.N.); hi-shiomi@hyo-med.ac.jp (H.S.); sh-fukunishi@hyo-med.ac.jp (S.F.); sh-shinzaki@hyo-med.ac.jp (S.S.); 2 Yoh Digestive Clinic, Wakayama 640-8269, Japan; 3 Department of Gastroenterology, Kano General Hospital, Osaka 531-0041, Japan; nishiguchi@heartfull.or.jp

**Keywords:** chronic liver disease, bioimpedance analysis, estimated glomerular filtration rate, ROC curve, grip strength, skeletal muscle mass index

## Abstract

**Background/Objectives:** Sarcopenia is an important clinical feature of patients with chronic liver disease (CLD). However, special devices are required to determine skeletal muscle mass. We evaluated the usefulness of body surface area (BSA) for estimating muscle mass and diagnosing sarcopenia in patients with CLD. **Methods:** We retrospectively studied 1889 Japanese patients with CLD who underwent bioimpedance analysis (BIA) (training cohort, n = 983; validation cohort, n = 906). The optimal cutoff values for predicting low skeletal muscle mass index (SMI) were determined using ROC analysis. We also assessed 1229 patients whose BSA and grip strength (GS) data were obtained on the same day and evaluated the diagnostic performance of the determined cutoff values of BSA for the diagnosis of sarcopenia. **Results:** In the training cohort, a strong correlation was observed between the SMI and BSA (r = 0.883, *p* < 0.0001). The cutoff values of BSA for predicting low SMI were 1.68 m^2^ for men and 1.48 m^2^ for women. Regarding the presence of low SMI, 776 (78.9%) and 730 (80.5%) patients were correctly diagnosed in the training and validation cohorts, respectively. The sensitivity and specificity of the combination of BSA and GS for sarcopenia were 82.7% and 97.1%, respectively, and 1175 patients (95.6%) were correctly diagnosed. **Conclusions:** BSA was highly correlated with SMI, suggesting that BSA could facilitate noninvasive estimation of low skeletal muscle mass in patients with CLD.

## 1. Introduction

Sarcopenia was proposed by Rosenberg in 1989 as a clinical condition characterized by age-related decline in muscle volume [1]. In relation to Rosenberg’s study, the idea of ‘secondary/disease-related sarcopenia’ was proposed, and patients with chronic liver disease (CLD) were subsequently considered to be at risk of developing secondary/disease-related sarcopenia [2,3,4,5,6,7]. Decreased muscle volume is essential for the diagnosis of sarcopenia, and skeletal muscle mass index (SMI), which is calculated as skeletal muscle mass/height^2^, is frequently used to determine low muscle volume. In the Asian Working Group on Sarcopenia (AWGS 2019) criteria [8] and the Japan Society of Hepatology guidelines for sarcopenia in chronic liver diseases [9,10], various techniques, including bioimpedance analysis (BIA) and computed tomography, are recommended for the diagnosis of low muscle volume. However, such specific devices are only available in a limited number of institutions, and a method that is able to easily estimate muscle volume without any special devices would be desirable.

Estimated glomerular filtration rate (eGFR) is well known to be influenced by skeletal muscle mass [11]. In clinical practice, the influence of skeletal muscle mass is adjusted by body surface area (BSA) [12], suggesting that BSA can represent skeletal muscle volume. In a study of 45 healthy adults, skeletal muscle area measured by computed tomography was suggested to be correlated with BSA [13]. Thus, we hypothesized that BSA could help estimate muscle volume in patients with CLD. However, the relationship between BSA data and muscle volume measured using BIA is unknown. In addition, the impact of BSA data on the diagnosis of sarcopenia in patients with CLD has not been investigated. This study aimed to evaluate the clinical utility of BSA data for the diagnosis of low SMI and sarcopenia in patients with CLD.

## 2. Patients and Methods

### 2.1. Patients

**Figure 1:**
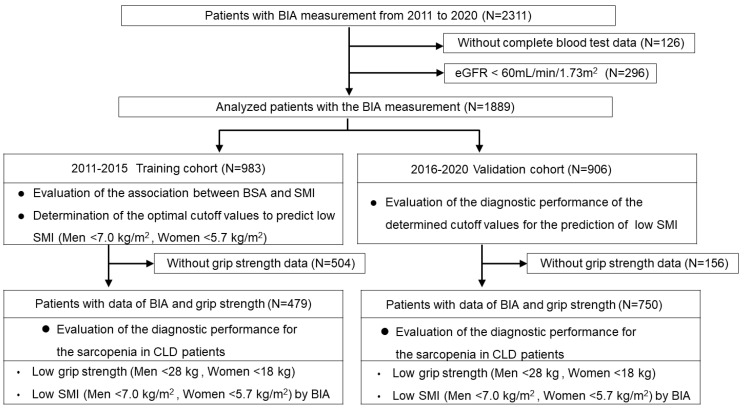
Flowchart of the study. We retrospectively studied 1889 Japanese patients with CLD who underwent bioimpedance analysis (BIA). We used the BIA data from the training cohort (n = 983) to analyze the association between BSA and SMI values and determined the optimal cutoff values to predict low SMI according to receiver operating characteristic (ROC) curves. We then assessed the validation cohort (n = 906) and evaluated the utility of the cutoff values for the prediction of low SMI. We also assessed 1229 patients with BSA and grip strength (GS) data and evaluated their diagnostic performance for sarcopenia.

CLD patients whose BIA data were obtained from August 2011 to December were consecutively enrolled (n = 2311). BIA was not conducted on patients with the following criteria: (i) patients who were unable to maintain the measurement posture, (ii) patients with a cardiac pacemaker, (iii) patients with clinically significant ascites detected by physical examination (comparable to ascites grade 2 or 3 in the International Ascites Club grading system [14]), (iv) patients with artificial joints, and (v) patients who were pregnant or with suspected pregnancy. Patients without complete blood test data were excluded from this study (n = 126). Patients without normal renal function (patients whose eGFR values were less than 60 mL/min/1.73 m^2^) [12,15] were also excluded (n = 296), as the presence of chronic kidney disease could have potentially been related to disease-related sarcopenia [6]. The basic characteristics of the 1889 patients in which BIA data were analyzed (see Figure 1) are shown in Table 1. The diagnosis of liver cirrhosis (LC) was determined according to current guidelines [16]. In the present study, the BSA values of the patients were determined using the commonly used Du Bois formula (height (cm) ^0.725^ × weight (kg) ^0.425^ × 0.007184) [17]. In addition, we calculated the geriatric nutritional risk index (GNRI), which has been used for nutritional assessment of patients with various diseases, including liver disease [18,19,20,21].

### 2.2. Diagnosis of Low SMI and Sarcopenia

The presence of low SMI and sarcopenia was diagnosed according to the guidelines of the Japan Society of Hepatology [9], which were determined based on a previous multicenter study [10] and are consistent with the AWGS 2019 criteria [8]. Body composition was assessed by the BIA method using the InBody 770 device (InBody Japan Inc., Tokyo, Japan), and SMI was calculated as total appendicular skeletal muscle (kg)/height (m)^2^. Low SMI was defined as SMI <7.0 kg/m^2^ (men) and <5.7 kg/m^2^ (women). Low grip strength (GS) was defined as GS <28 kg (men) and <18 kg (women). Patients with sarcopenia were defined as those with both low GS and low SMI [8,9].

Among a total of 1889 patients analyzed (see Figure 1), we used the BIA data of 983 patients obtained from August 2011 to December 2015 as the training cohort to analyze the association between BSA and SMI values. We determined the optimal cutoff values to predict low SMI according to receiver operating characteristic (ROC) curves. We then analyzed the patients who underwent BIA analyses from January 2016 to December 2020 (n = 906) and evaluated the utility of the cutoff values for the prediction of low SMI (see Figure 1).

Of the 1889 patients in which BIA data were analyzed, GS was also measured on the same day in 1229 patients. We evaluated the utility of the determined cutoff values for the diagnosis of sarcopenia, defined by the presence of low GS and low SMI (see Figure 1). Since the reference values for the diagnosis of sarcopenia differ by sex [8,16], optimal cutoff values were determined for each sex. Therefore, the diagnostic performance of each sex was evaluated as primary data in this study. In addition, we conducted sub-analyses based on age, etiology, and body mass index (BMI), which were considered to influence the data in relation to the diagnosis of sarcopenia.

### 2.3. Statistical Analyses

For continuous variables, the Mann–Whitney U test was used to assess differences between 2 groups. For categorical data, Fisher’s exact test or the chi-squared test were used to evaluate the statistical differences between the 2 groups. Pearson’s correlation coefficient (r) was used to analyze the correlation between 2 parameters. Statistical analyses were conducted using the JMP 17.2 software program (SAS Institute Inc. Cary, NC, USA). A *p* value of <0.05 was considered to indicate statistical significance.

The study protocol conformed to the 1964 Declaration of Helsinki and its later amendments, and the study was approved by the institutional review board of our hospital (Approval No. 1831 and 3431). Due to the retrospective study design, an opt-out method was used in this study.

## 3. Results

### 3.1. Characteristics of the Training Cohort

The basic characteristics of the training cohort (n = 983) used to analyze the association between BSA values and SMI values are shown in Table 1. The median age was 62 years, and 497 patients (50.6%) were male patients. Two hundred and fifty-seven patients (26.1%) had LC. The number of patients with albumin–bilirubin (ALBI) grades 1, 2, and 3 were 578 (58.8%), 369 (37.5%), and 36 (3.7%), respectively. A total of 348 patients (35.4%) had low SMI, including 153 male patients (153/497, 30.8%) and 195 female patients (195/486, 40.1%).

### 3.2. Determination of the Cutoff Values of BSA to Predict Low SMI, and Their Diagnostic Performance

**Figure 2:**
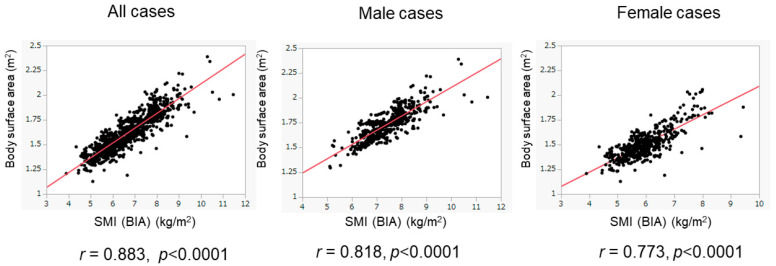
Correlation of BSA values with SMI values in the training cohort. The correlation between BSA and SMI values was evaluated in the training cohort (n = 983). BSA values were well correlated with SMI values. The results of the overall population (**left** panel), male patients (**middle** panel), and female patients (**right** panel) are shown.

**Figure 3:**
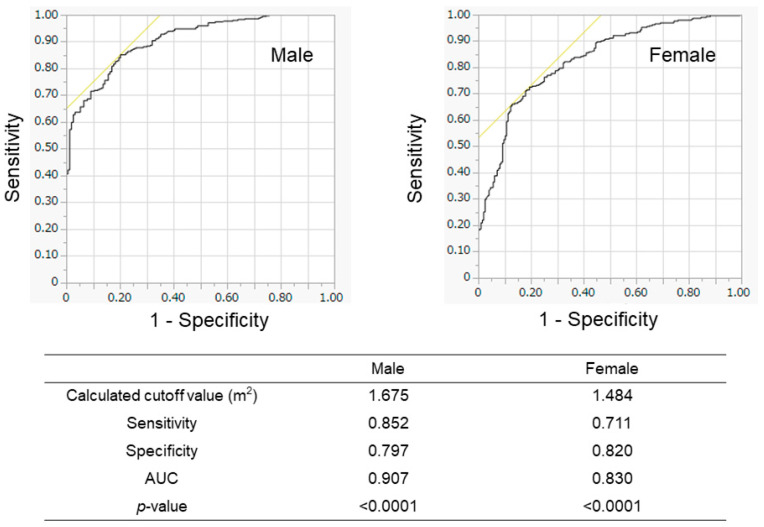
Receiver operating characteristic (ROC) analyses for the prediction of low SMI according to BSA in the training cohort. The results of male patients (**left** panel) and female patients (**right** panel) are shown. The calculated optimal cutoff values were 1.675 m^2^ for men and 1.484 m^2^ for women. AUROC—area under the ROC curve.

We evaluated the association between BSA and SMI in the training cohort (Figure 2). The BSA values correlated well with the SMI values in all patients (*r* = 0.883), in male patients (*r* = 0.818), and in female patients (*r* = 0.773). When we conducted the ROC analyses, the cutoff values of BSA to suggest the presence of low SMI were 1.675 m^2^ for male patients and 1.484 m^2^ for female patients (Figure 3). The AUROCs (areas under the ROC curve) for predicting low SMI were 0.907 (sensitivity—85.2%; specificity—79.7%) for male patients and 0.830 (sensitivity—71.1%; specificity—82.0%) for female patients. According to the abovementioned results, the cutoff values to predict low SMI were 1.68 m^2^ for male patients and 1.48 m^2^ for female patients. The diagnostic performance of the determined cutoff values for detecting low SMI is shown in Table 2. Regarding the training cohort, the sensitivity and specificity for the detection of low SMI in all patients were 80.5% and 78.1%, respectively, and 776 of the 983 patients were correctly diagnosed (diagnostic accuracy—78.9%). Our determined cutoff values showed 80.4% sensitivity and 83.4% specificity in male patients (diagnostic accuracy—82.5%), and 80.5% sensitivity and 71.8% specificity in female patients (diagnostic accuracy—75.3%).

### 3.3. Characteristics of the Validation Cohort and Diagnostic Performance of BSA for Low SMI

**Figure 4:**
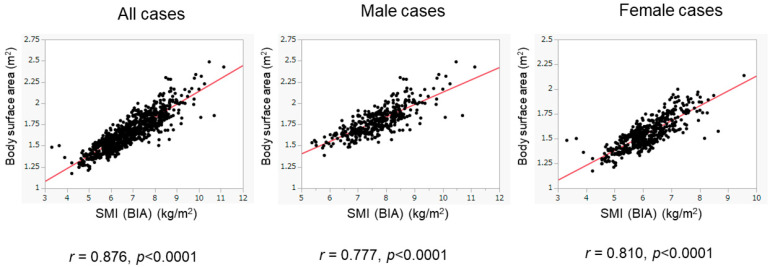
Correlation of BSA with SMI in the validation cohort. The correlation between BSA and SMI was evaluated in the validation cohort (n = 906). BSA was well correlated with SMI. The results of the overall population (left panel), the male patients (middle panel), and the female patients (right panel) are shown.

We then assessed patients whose BIA data were obtained from 2016 to 2020 as the validation cohort. The basic characteristics of the validation cohort are presented in Table 1. The validation cohort included 416 male patients (45.9%), with a median age of 61 years. There were 227 patients (25.1%) with low SMI, including 86 male patients (86/416, 20.7%) and 141 female patients (141/490, 28.8%). There were 716 patients (79.0%) with ALBI grade 1, 173 (19.1%) with ALBI grade 2, and 17 (1.9%) with ALBI grade 3. Despite the presence of differences in various clinical variables between the training and validation cohorts, the BSA values were positively and highly correlated with BIA-based SMI values in the validation cohort (overall population: *r* = 0.876, *p* < 0.0001; male patients: *r* = 0.777, *p* < 0.0001; female patients: *r* = 0.810, *p* < 0.0001) (Figure 4). The diagnostic performance of our determined cutoff values (1.68 m^2^ for men and 1.48 m^2^ for women) to detect low SMI in the validation cohort is shown in Table 3. Regarding the validation cohort, the sensitivity and specificity for the overall population were 70.5% and 83.9%, respectively, and 730 of 906 patients were correctly diagnosed (diagnostic accuracy—80.5%). Our determined cutoff values showed 75.6% sensitivity and 87.6% specificity in male patients (diagnostic accuracy—85.0%), and 67.4% sensitivity and 80.5% specificity in female patients (diagnostic accuracy—76.7%).

### 3.4. Usefulness of the Cutoff Values of BSA for the Diagnosis of Sarcopenia

To evaluate the utility of the determined BSA cutoff values for the diagnosis of sarcopenia, 1229 patients whose BSA and GS data were obtained on the same day were analyzed (Figure 1). The basic characteristics of the study participants are shown in Table 4.

The median age of the cohort was 62 years, and 578 patients were male. There were 920 patients (74.9%) with ALBI grade 1, 291 (23.7%) with ALBI grade 2, and 18 (1.5%) with ALBI grade 3. There were 127 patients (10.3%) with sarcopenia, including 51 male patients (51/578, 8.8%) and 76 female patients (76/651, 11.7%). When we assessed the data of the overall population, our BSA cutoff values (<1.68 m^2^ for men and <1.48 m^2^ for women) combined with the GS cutoff values (<28 kg for men and <18 kg for women) showed 82.7% sensitivity and 97.1% specificity in the overall population (diagnostic accuracy—95.6%), 80.4% sensitivity and 97.5% specificity in male patients (diagnostic accuracy—96.0%), and 84.2% sensitivity and 96.7% specificity in female patients (diagnostic accuracy—95.2%) (Table 5).

We divided the patients into two groups according to the year in which the data were obtained (2011–2015 and 2016–2020). When we compared the clinical variables between the two groups, significant differences were identified in various clinical parameters. In addition, in male patients, the ratios of low SMI presence/absence and sarcopenia presence/absence differed between the two groups. In female patients, the GS values also showed a significant difference between the two groups (Table 4). We further evaluated whether the diagnostic performance of our criteria (the combination of BSA and GS) would be conserved in the two groups with different clinical characteristics.

Regarding the patients whose data were obtained from 2010 to 2015, the diagnostic performance of the combination of BSA and GS for sarcopenia was as follows: overall population—83.6% sensitivity and 96.4% specificity (diagnostic accuracy—94.6%); male patients—78.6% sensitivity and 97.0% specificity (diagnostic accuracy—94.8%); and female patients—87.2% sensitivity and 95.7% specificity (diagnostic accuracy—94.4%) (Table 6).

Regarding the patients whose data were obtained from 2016 to 2020, the diagnostic performance of the combination of BSA and GS for sarcopenia was as follows: overall population—81.7% sensitivity and 97.5% specificity (diagnostic accuracy—96.3%); male patients—82.6% sensitivity and 97.9% specificity (diagnostic accuracy—96.8%); and female patients—81.1% sensitivity and 97.3% specificity (diagnostic accuracy—95.8%) (Table 7).

### 3.5. Sub-Analyses of the Diagnostic Performance of BSA for Low SMI and Sarcopenia

The reference values for the diagnosis of sarcopenia differed by sex [8], so we determined the optimal cutoff values and evaluated the diagnostic performance for each sex. We additionally conducted sub-analyses based on age, etiology, and body mass index (BMI), which were considered to be related to the diagnosis of sarcopenia. We examined whether or not there were differences in diagnostic performance according to age (≥65 vs. <65 years old), etiology (viral vs. nonviral), and BMI (≥23 vs. <23 kg/m^2^). Regarding the prediction for low SMI, the sensitivity was low, but specificity was high in patients with high BMIs (≥23 kg/m^2^) or in non-elderly patients (<65 years old) (Table 2 and Table 3). Similarly, regarding the diagnostic performance of sarcopenia, lower sensitivity was observed in patients with high BMIs (≥23 kg/m^2^) or in non-elderly patients (<65 years), regardless of the study period, although the number of sarcopenic patients with such characteristics (high BMI or non-elderly) was relatively small (Table 5, Table 6 and Table 7).

## 4. Discussion

More than 30 years have passed since the concept of sarcopenia was first proposed [1]. Recently, sarcopenia has received much attention in the field of public health because of its close association with the development of unfavorable clinical events, including falls, fractures, infections, frailty, and poor survival rates [22,23,24,25,26]. The evaluation of skeletal muscle volume is essential to the diagnosis of sarcopenia; however, special devices to determine muscle volume, such as the BIA method and dual-energy X-ray absorptiometry (DEXA), are only available in a limited number of institutions [8].

For the estimation of renal function, BSA was used to adjust for the influence of muscle mass volume. Thus, we considered that BSA could be used to noninvasively assess skeletal muscle mass. Accordingly, the current study was planned with a large number of patients. In the present study, we showed that BSA values were highly correlated with BIA-based SMI values, suggesting that BSA value could be a simple tool that could be applied to screen for sarcopenia in patients with CLD.

In this study, we used ROC analysis to determine the cutoff values of BSA to predict low skeletal muscle mass (Figure 4). The cutoff values obtained from the regression line to predict low SMI were 1.68 m^2^ for males and 1.47 m^2^ for females in the training cohort (Supplementary Figure S1). In addition, the cutoff values, which were obtained from the regression line in the validation group, were 1.69 m^2^ for male patients and 1.49 m^2^ for female patients (Supplementary Figure S2). These cutoff values were similar to those determined by ROC analysis in this study. Recently, the situation of liver disease in Japan has changed remarkably due to advances in antiviral treatments and changes in lifestyle [27], and the patient characteristics in the study were different between the groups whose data were obtained in different periods, such as the increased ratios of nonviral etiologies in recent years (Table 1 and Table 4). However, the BSA showed similar diagnostic performance regardless of the period in which the data were obtained (Table 2, Table 3, Table 5, Table 6 and Table 7). These findings suggest that BSA values are highly correlated with BIA-measured SMI values and could be helpful in predicting SMI in various types of CLDs. Thus, our study suggests that BSA cutoff values could be used as a simple screening tool for predicting low SMI and sarcopenia without using any special equipment. Recently, the finger-ring (Yubi-wakka) test, which can be performed without any special tools, has been reported to be an easy and noninvasive screening method for sarcopenia [28]. A comparison of the diagnostic performance between the finger-ring method and the BSA-based method may be interesting. In addition, the combined use of BSA and finger-ring methods may also be interesting.

Our cutoff values for BSA showed moderate diagnostic ability in the prediction of low SMI (Table 2 and Table 3). In addition, combined with the presence of low GS, the presence of low SMI estimated by BSA showed good diagnostic performance, with high specificity for sarcopenia (Table 5, Table 6 and Table 7). Regarding the diagnostic performance of the GS alone for the prediction of sarcopenia, its sensitivity and negative predictive value were 100%, since GS value was used as one of the diagnostic criteria for sarcopenia; however, its specificity and diagnostic accuracy were relatively low (Supplementary Tables S1–S3). Thus, the high sensitivity and negative predictive value of GS were suggested to contribute to the improved diagnostic performance of the combination of GS and BSA, as compared to BSA alone. Sarcopenia was originally defined as an age-related loss of muscle mass [1], and research regarding sarcopenia has classically focused on decreased skeletal muscle volume rather than parameters that reflect muscle function, such as GS [29,30]. However, subsequent studies have used the term ‘sarcopenia’ to describe a more comprehensive idea that includes muscle strength decline [31]. We previously reported the importance of grip strength in complex liver events in patients with CLD [32]. In addition, Hanai et al. reported that handgrip strength decline was significantly associated with increased mortality risk in patients with LC [33]. This study also showed the importance of GS measurements for the correct diagnosis of sarcopenia. Longitudinal studies have shown that muscle strength declines much faster than muscle mass in older adults, suggesting the importance of muscle strength for muscle function [34].

The present study has several limitations. First, this was a cross-sectional study conducted at a single institution, with a retrospective study design. Second, the analyzed cohort mainly comprised HCV-infected Japanese patients with normal renal function. Third, as suggested by the sub-analyses, the diagnostic performance of BSA could be influenced by factors such as BMI and age. Thus, the diagnostic performance for some specific patients, such as those with ‘sarcopenic obesity’ or ‘non-elderly sarcopenia,’ might be insufficient, although the number of such cases was relatively small. Because of the inadequate data collection of the metabolism-associated variables, we could not determine the actual number of patients with dysmetabolic etiology. However, it would be interesting to know how many patients actually had dysmetabolic etiology and to know the BMI and diagnostic performance of BSA alone in this subgroup, since in Western countries dysmetabolic etiology currently represents the main cause of liver disease and widespread obesity represents an obstacle to the application of BSA. Fourth, there were several differences between the background characteristics of the training and validation groups.

In summary, we showed that BSA values were highly correlated with BIA-based SMI values. We determined the optimal cutoff values to predict low skeletal muscle mass in Japanese patients with CLD. Our results suggest that BSA could help with noninvasive assessment for sarcopenia in CLD patients.

## Figures and Tables

**Table 1:** Patient characteristics used to evaluate the associations between BSA values and SMI values.

Variables	All Cases (n = 1889)	Training Cohort (n = 983)	Validation Cohort (n = 906)	*p*-Value
Age (years)	61 (51.5–69)	62 (53–69)	61 (51–69)	0.1945
Gender, male/female	976/913	497/486	416/490	0.0436
Etiology of the liver diseases; HCV/HBV/others	1025/189/675	654/81/248	371/108/427	<0.0001
Presence of liver cirrhosis, yes/no	424/1465	257/726	167/739	<0.0001
Body mass index (kg/m^2^)	22.9 (20.5–25.7)	22.1 (20.2–25.0)	23.7 (21.2–26.5)	<0.0001
Nutrition-related risk (GNRI):				<0.0001
Major (<82)	52 (2.8%)	27 (2.7%)	25 (2.8%)	
Moderate (82 to <92)	182 (9.6%)	145 (14.8%)	37 (4.1%)	
Low (92 to <98)	229 (12.1%)	156 (15.9%)	73 (8.1%)	
Absent (≥98)	1426 (75.5%)	655 (66.6%)	771 (85.1%)	
AST (U/L)	26 (20–38)	28 (22–43)	24 (19–32.3)	<0.0001
ALT (U/L)	23 (15–37)	24 (16–42)	21 (15–33)	0.0001
γ-GTP (U/L)	28 (18–49)	30 (19–50)	25 (17–47)	0.001
Serum albumin (g/dL)	4.2 (3.8–4.4)	4.0 (3.7–4.4)	4.3 (4.0–4.5)	<0.0001
Total bilirubin (mg/dL)	0.8 (0.6–1.1)	0.9 (0.7–1.2)	0.8 (0.6–1.0)	<0.0001
eGFR (ml/min/1.73m^2^)	85.3 (74.1–99.6)	86.6 (75.7–100.8)	83.6 (72.7–98.3)	0.0004
Hb (g/dL)	13.3 (12.1–14.5)	13.0 (11.6–14.2)	13.6 (12.6–14.7)	<0.0001
PT (%)	90.1 (78.2–99.6)	84.7 (72.1–94.7)	95.0 (86.0–103.5)	<0.0001
Platelet count (×10^3^/mm^3^)	168 (112–221)	141 (93–196)	194 (144–242)	<0.0001
ALBI score	−2.79 (−3.04 to −2.45)	−2.70 (−2.94 to −2.28)	−2.91 (−3.10 to −2.65)	<0.0001
ALBI grade, 1/2/3	1294/542/53	578/369/36	716/173/17	<0.0001
SMI (kg/m^2^), male	7.63 (6.97–8.14)	7.50 (6.86–7.99)	7.70 (7.08–8.27)	<0.0001
SMI (kg/m^2^), female	5.95 (5.52–6.47)	5.83 (5.45–6.33)	6.06 (5.65–6.62)	<0.0001
Presence of low SMI, n (%), male	239 (26.2%)	153 (30.8%)	86 (20.7%)	0.0005
Presence of low SMI, n (%), female	336 (34.4%)	195 (40.1%)	141 (28.8%)	0.0002
Body surface area (m^2^), male	1.75 (1.65–1.87)	1.74 (1.63–1.85)	1.78 (1.68–1.88)	<0.0001
Body surface area (m^2^), female	1.51 (1.42–1.60)	1.48 (1.40–1.56)	1.53 (1.44–1.63)	0.0003

Body surface area was determined by the Du Bois Method. Data are expressed as median (interquartile range). The *p*-values represent the differences between the training cohort and the validation cohort. BSA—body surface area; SMI—skeletal muscle mass index; HCV—hepatitis C virus; HBV—hepatitis B virus; GNRI—geriatric nutritional risk index; AST—aspartate aminotransferase; ALT—alanine aminotransferase; γ-GTP—γ-glutamyl transpeptidase; eGFR—estimated glomerular filtration rate; Hb—hemoglobin; PT—prothrombin time; ALBI—albumin–bilirubin; SMI—skeletal muscle index.

**Table 2:** Diagnostic performance of BSA values for the prediction of BIA-measured low SMI in the training cohort.

Training Cohort		Sensitivity	Specificity	PPV	NPV	Diagnostic Accuracy
All cases (N = 983, 100%)		280/348 (80.5%)	496/635 (78.1%)	280/419 (66.8%)	496/564 (87.9%)	776/983 (78.9%)
Gender	Male (N = 497, 50.6%)	123/153 (80.4%)	287/344 (83.4%)	123/180 (68.3%)	287/317 (90.5%)	410/497 (82.5%)
	Female (N = 486, 49.4%)	157/195 (80.5%)	209/291 (71.8%)	157/239 (65.7%)	209/247 (84.6%)	366/486 (75.3%)
Age (-years old)	≥65 (N = 407, 41.4%)	173/201 (86.1%)	134/206 (65.0%)	173/245 (70.6%)	134/162 (82.7%)	307/407 (75.4%)
	<65 (N = 576, 58.6%)	107/147 (72.8%)	362/429 (84.4%)	107/174 (61.5%)	362/402 (90.0%)	469/576 (81.4%)
Etiology	Viral (N = 735, 74.8%)	207/262 (79.0%)	356/473 (75.3%)	207/324 (63.9%)	356/411 (86.6%)	563/735 (76.6%)
	Nonviral (N = 248, 25.2%)	73/86 (84.9%)	140/162 (86.4%)	73/95 (76.8%)	140/153 (91.5%)	213/248 (85.9%)
BMI (kg/m^2^)	≥23 (N = 418, 42.5%)	23/44 (52.3%)	344/374 (92.0%)	23/53 (43.4%)	344/365 (94.2%)	367/418 (87.8%)
	<23 (N = 565, 57.5%)	257/304 (84.5%)	152/261 (58.2%)	257/366 (70.2%)	152/199 (76.4%)	409/565 (72.4%)

PPV—positive predictive value, NPV—negative predictive value, BMI—body mass index.

**Table 3:** Diagnostic performance of BSA values for the prediction of BIA-measured low SMI in the validation cohort.

Validation Cohort		Sensitivity	Specificity	PPV	NPV	Diagnostic Accuracy
All cases (N = 906, 100%)		160/227 (70.5%)	570/679 (83.9%)	160/269 (59.5%)	570/637 (89.5%)	730/906 (80.5%)
Gender	Male (N = 416, 45.9%)	65/86 (75.6%)	289/330 (87.6%)	65/106 (61.3%)	289/310 (93.2%)	354/416 (85.0%)
	Female (N = 490, 54.1%)	95/141 (67.4%)	281/349 (80.5%)	95/163 (58.3%)	281/327 (85.9%)	376/490 (76.7%)
Age (-years old)	≥65 (N = 376, 41.5%)	117/152 (77.0%)	175/224 (78.1%)	117/166 (70.5%)	175/210 (83.3%)	292/376 (77.7%)
	<65 (N = 530, 58.5%)	107/147 (72.8%)	362/429 (84.4%)	107/174 (61.5%)	362/402 (90.0%)	469/576 (81.4%)
Etiology	Viral (N = 479, 52.9%)	103/145 (71.0%)	283/334 (84.7%)	103/154 (66.9%)	283/325 (87.1%)	386/479 (80.6%)
	Nonviral (N = 427, 47.1%)	57/82 (69.5%)	287/345 (83.2%)	57/115 (49.6%)	287/312 (92.0%)	344/427 (80.6%)
BMI (kg/m^2^)	≥23 (N = 512, 56.5%)	16/45 (35.6%)	437/467 (93.6%)	16/46 (34.8%)	437/466 (93.8%)	453/512 (88.5%)
	<23 (N = 394, 43.5%)	144/182 (79.1%)	133/212 (62.7%)	144/223 (64.6%)	133/171 (77.8%)	277/394 (70.3%)

PPV—positive predictive value, NPV—negative predictive value, BMI—body mass index.

**Table 4:** Patient characteristics for the prediction of sarcopenia.

Variables	All Cases (n = 1229)	Cases 2011–2015 (n = 479)	Cases 2016–2020 (n = 750)	*p*-Value
Age (years)	62 (52–70)	64 (55–70)	61 (51–69)	0.1078
Gender, male/female	578/651	230/249	348/402	0.5798
Etiology of the liver diseases: HCV/HBV/others	764/116/349	426/24/29	338/92/320	<0.0001
Presence of liver cirrhosis, yes/no	260/969	110/369	150/600	0.2162
Body mass index (kg/m^2^)	22.6 (20.4–25.5)	21.6 (19.8–24.0)	23.5 (21.0–26.1)	<0.0001
Nutrition-related risk (GNRI):				<0.0001
Major (<82)	28 (2.3%)	7 (1.5%)	21 (2.8%)	
Moderate (82 to <92)	77 (6.3%)	44 (9.2%)	33 (4.4%)	
Low (92 to <98)	141 (11.5%)	75 (15.7%)	66 (8.8%)	
Absent (≥98)	983 (80.0%)	353 (73.7%)	630 (84.0%)	
AST (U/L)	24 (19–34)	26 (21–37)	23 (19–32)	<0.0001
ALT (U/L)	20 (14–33)	20 (14–36)	20 (14–32)	0.8822
γ-GTP (U/L)	25 (17–44)	25 (17–43)	24 (17–46)	0.9600
Serum albumin (g/dL)	4.2 (3.9–4.5)	4.1 (3.9–4.4)	4.3 (4.0–4.5)	<0.0001
Total bilirubin (mg/dL)	0.8 (0.6–1.0)	0.8 (0.6–1.1)	0.8 (0.6–1.0)	0.0649
eGFR (ml/min/1.73m^2^)	83.7 (73.1–98.0)	86.1 (75.6–99.7)	82.1 (71.8–96.2)	<0.0001
Hb (g/dL)	13.3 (12.1–14.5)	13.0 (11.6–14.2)	13.5 (12.6–14.7)	<0.0001
PT (%)	91.6 (82.4–100.9)	87.8 (80.2–95.2)	94.4 (85.6–103.1)	<0.0001
Platelet count (×10^3^/mm^3^)	172 (124–219)	14.4 (10.9–19.3)	18.8 (13.8–23.2)	<0.0001
ALBI score	−2.84 (−3.07 to −2.59)	−2.76 (−2.98 to −2.53)	−2.91 (−3.10 to −2.65)	<0.0001
ALBI grade, 1/2/3	920/291/18	335/139/5	585/152/13	0.0017
SMI (kg/m^2^), male	7.64 (6.99–8.15)	7.41 (6.82–7.97)	7.70 (7.08–8.25)	0.0002
SMI (kg/m^2^), female	5.90 (5.53–6.41)	5.77 (5.42–6.06)	6.04 (5.60–6.62)	<0.0001
Presence of low SMI, n (%), male	145 (25.1%)	72 (31.3%)	73 (21.0%)	0.0053
Presence of low SMI, n (%), female	220 (33.8%)	102 (41.0%)	118 (29.4%)	0.0024
Body surface area (m^2^), male	1.75 (1.65–1.86)	1.74 (1.63–1.83)	1.78 (1.67–1.87)	0.0002
Body surface area (m^2^), female	1.51 (1.41–1.59)	1.47 (1.39–1.55)	1.53 (1.44–1.62)	<0.0001
Grip strength (kg), male	35.5 (29.9–41.6)	34.5 (29.0–41.3)	36.1 (30.5–41.6)	0.1250
Grip strength (kg), female	21.1 (18.3–23.8)	20.4 (17.2–23.3)	21.3 (18.8–24.3)	0.0002
Presence of sarcopenia (%), male	51 (8.8%)	28 (12.2%)	23 (6.6%)	0.0225
Presence of sarcopenia (%), female	76 (11.7%)	39 (15.7%)	37 (9.2%)	0.0138

Body surface area was determined by the Du Bois Method. Data are expressed as median (interquartile range). The *p*-values represent the differences between the training cohort and the validation cohort. BSA—body surface area; SMI—skeletal muscle mass index; HCV—hepatitis C virus; HBV—hepatitis B virus; GNRI—geriatric nutritional risk index; AST—aspartate aminotransferase; ALT—alanine aminotransferase; γ-GTP—γ-glutamyl transpeptidase; eGFR—estimated glomerular filtration rate; Hb—hemoglobin; PT—prothrombin time; ALBI—albumin–bilirubin; SMI—skeletal muscle index.

**Table 5:** Diagnostic performance of the combination of BSA value and the grip strength for the prediction of sarcopenia (data obtained from 2011 to 2020).

Validation Cohort		Sensitivity	Specificity	PPV	NPV	Diagnostic Accuracy
All cases from 2011 to 2020 (N = 1229, 100%)		105/127 (82.7%)	1070/1102 (97.1%)	105/137 (76.6%)	1070/1092 (98.0%)	1175/1229 (95.6%)
Gender	Male (N = 578, 47.0%)	41/51 (80.4%)	514/527 (97.5%)	41/54 (75.9%)	514/524 (98.1%)	555/578 (96.0%)
	Female (N = 651, 53.0%)	64/76 (84.2%)	556/575 (96.7%)	64/83 (77.1%)	556/568 (97.9%)	620/651 (95.2%)
Age (-years old)	≥65 (N = 544, 44.3%)	86/95 (90.5%)	422/449 (94.0%)	86/113 (76.1%)	422/431 (97.9%)	508/544 (93.4%)
	<65 (N = 685, 55.7%)	19/32 (59.4%)	648/653 (99.2%)	19/24 (79.2%)	648/661 (98.0%)	667/685 (97.4%)
Etiology	Viral (N = 880, 71.6%)	81/100 (81.0%)	759/780 (97.3%)	81/102 (79.4%)	579/778 (97.6%)	840/880 (95.5%)
	Nonviral (N = 349, 28.4%)	24/27 (88.9%)	311/322 (96.6%)	24/35 (68.6%)	311/314 (99.0%)	335/349 (96.0%)
BMI (kg/m^2^)	≥23 (N = 570, 46.4%)	7/16 (43.8%)	541/554 (97.7%)	7/20 (35.0%)	541/550 (98.4%)	548/570 (96.1%)
	<23 (N = 659, 53.6%)	98/111 (88.3%)	529548 (96.5%)	98/117 (83.8%)	529/542 (97.6%)	627/659 (95.1%)

PPV—positive predictive value; NPV—negative predictive value; BMI—body mass index.

**Table 6:** Diagnostic performance of the combination of BSA value and the grip strength for the prediction of sarcopenia (data obtained from 2011 to 2015).

Validation Cohort		Sensitivity	Specificity	PPV	NPV	Diagnostic Accuracy
All cases from 2011 to 2015 (N = 479, 100%)		56/67 (83.6%)	397/412 (96.4%)	56/71 (83.6%)	397/408 (97.3%)	453/479 (94.6%)
Gender	Male cases (N = 230, 48.0%)	22/28 (78.6%)	196/202 (97.0%)	22/28 (78.6%)	196/202 (97.0%)	218/230 (94.8%)
	Female cases (N = 249, 52.0%)	34/39 (87.2%)	201/210 (95.7%)	34/43 (79.0%)	201/206 (97.6%)	235/249 (94.4%)
Age (-years old)	≥65 (N = 219, 45.7%)	41/44 (93.2%)	163/175 (93.1%)	41/53 (77.4%)	163/166 (98.2%)	204/219 (93.2%)
	<65 (N = 260, 54.3%)	15/23 (65.2%)	234/237 (98.7%)	15/18 (83.3%)	234/242 (96.7%)	249/260 (95.8%)
Etiology	Viral (N = 450, 93.9%)	52/63 (82.5%)	373/387 (96.4%)	52/66 (78.8%)	373/384 (97.1%)	425/450 (94.4%)
	Nonviral (N = 29, 6.1%)	4/4 (100%)	24/25 (96.0%)	4/5 (80.0%)	24/24 (100%)	28/29 (96.6%)
BMI (kg/m^2^)	≥23 (N = 162, 33.8%)	2/6 (33.3%)	149/156 (95.5%)	2/9 (22.2%)	149/153 (97.4%)	151/162 (93.2%)
	<23 (N = 317, 66.2%)	54/61 (88.5%)	248/256 (96.9%)	54/62 (87.1%)	248/255 (97.3%)	302/317 (95.3%)

PPV—positive predictive value; NPV—negative predictive value; BMI—body mass index.

**Table 7:** Diagnostic performance of the combination of the BSA value and the grip strength for the prediction of sarcopenia (data obtained from 2016 to 2020).

Validation Cohort		Sensitivity	Specificity	PPV	NPV	Diagnostic Accuracy
All cases from 2016 to 2020 (N = 750, 100%)		49/60 (81.7%)	673/690 (97.5%)	49/66 (74.2%)	673/684 (98.4%)	722/750 (96.3%)
Gender	Male cases (N = 348, 46.4%)	19/23 (82.6%)	318/325 (97.9%)	19/26 (73.1%)	318/322 (98.8%)	337/348 (96.8%)
	Female cases (N = 402, 53.6%)	30/37 (81.1%)	355/365 (97.3%)	30/40 (75.0%)	355/362 (98.1%)	385/402 (95.8%)
Age (-years old)	≥65 (N = 325, 43.3%)	45/51 (88.2%)	259/274 (94.5%)	45/60 (75.0%)	259/265 (97.7%)	304/325 (93.5%)
	<65 (N = 425, 56.7%)	4/9 (44.4%)	414/416 (99.5%)	4/6 (66.7%)	414/419 (98.8%)	418/425 (98.4%)
Etiology	Viral (N = 430, 57.3%)	29/37 (78.4%)	386/393 (98.2%)	29/36 (80.6%)	386/394 (98.0%)	415/430 (96.5%)
	Nonviral (N = 320, 42.7%)	20/23 (87.0%)	287/297 (96.6%)	20/30 (66.7%)	287/290 (99.0%)	307/320 (95.9%)
BMI (kg/m^2^)	≥23 (N = 408, 54.4%)	5/10 (50.0%)	392/398 (98.5%)	5/11 (45.5%)	392/397 (98.7%)	397/408 (97.3%)
	<23 (N = 342, 54.6%)	44/50 (88.0%)	281/292 (96.2%)	44/55 (80.0%)	281/287 (97.9%)	325/342 (95.0%)

PPV—positive predictive value; NPV—negative predictive value; BMI—body mass index.

## Data Availability

The data presented in this study can be provided by the corresponding author upon reasonable request.

## Supplementary Materials

The following supporting information can be downloaded at https://www.mdpi.com/article/10.3390/diagnostics15030263/s1, Supplementary Figure S1: Formula for simple linear regression in the training cohort; Figure S2: Formula for simple linear regression in the validation cohort; Table S1: Diagnostic performance of the grip strength for the prediction of sarcoprenia (Data obtained from 2011 to 2020); Table S2: Diagnostic performance of the grip strength for the prediction of sarcoprenia (Data obtained from 2011 to 2015); Table S3: Diagnostic performance of the grip strength for the prediction of sarcoprenia (Data obtained from 2016 to 2020) are available in the supporting information.
