# Supplementary material for: Possible Use of Body Surface Area Value for Estimating Skeletal Muscle Mass in Chronic Liver Disease
Source: Diagnostics (Basel). 2025 Jan 23;15(3):263. doi: 10.3390/diagnostics15030263 (PMC11817660; doi:10.3390/diagnostics15030263)
Supplement: Supplementary file 1 [file diagnostics-15-00263-s001.zip › diagnostics-3407427-supplementary.pdf]

**Supporting information**

**Supplementary Figure S1: Formula for simple linear regression in the training cohort**

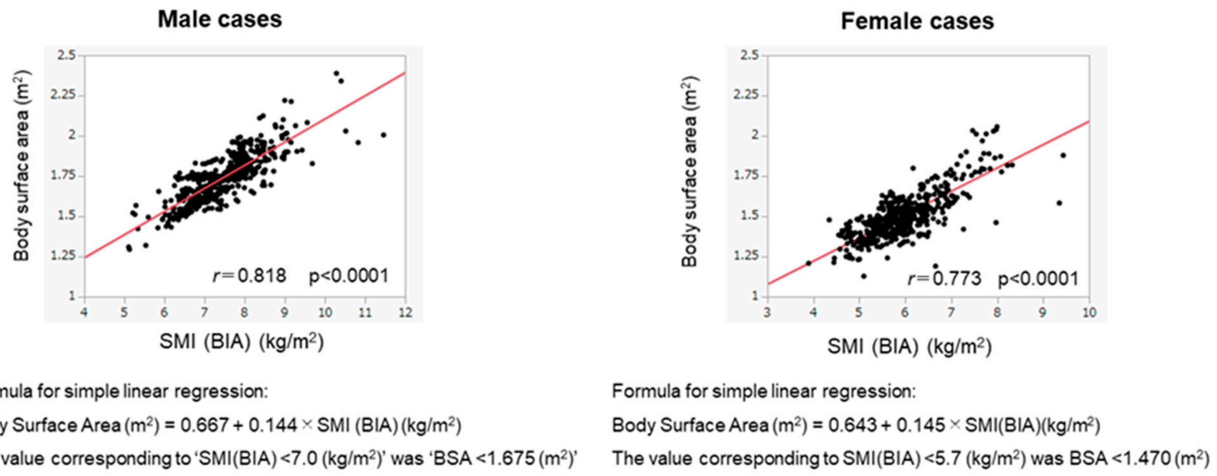


(Left panel) Formula for simple linear regression (male cases: n=497).

(Right panel) Formula for simple linear regression (female cases: n=486).

**Supplementary Figure S2: Formula for simple linear regression in the validation cohort**

**Supplementary Figure 2**

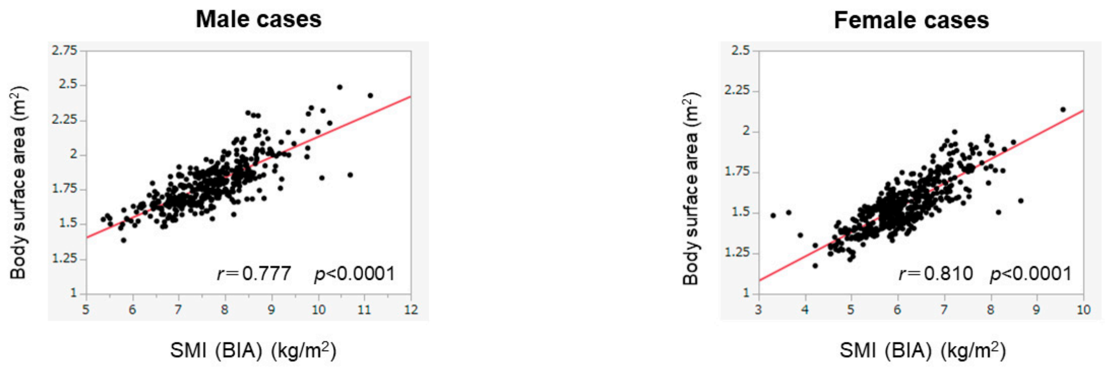


Formula for simple linear regression:
Body Surface Area ($m^2$) = 0.677 + 0.145 × SMI (BIA) (kg/$m^2$)
The value corresponding to 'SMI(BIA) < 7.0(kg/$m^2$)' was 'BSA < 1.692 ($m^2$)'

Formula for simple linear regression:
Body Surface Area ($m^2$) = 0.631 + 0.150 × SMI (BIA) (kg/$m^2$)
The value corresponding to 'SMI(BIA) <5.7 (kg/$m^2$)' was 'BSA < 1.486 ($m^2$)'

(Left panel) Formula for simple linear regression (male cases: n=416).

(Right panel) Formula for simple linear regression (female cases: n=490).

**Supplementary Table S1.** Diagnostic performance of the grip strength for the prediction of sarcoprenia (Data obtained from 2011 to 2020)

| Validation cohort | | Sensitivity | Specificity | PPV | NPV | Diagnostic accuracy |
|---|---|---|---|---|---|---|
| All cases from 2011 to 2020 (N=1229, 100%) | | 127/127(100%) | 977/1102 (88.7%) | 127/252 (50.4%) | 977/977 (100%) | 1104/ 1229 (89.8%) |
| Gender | Male (N=578, 47.0%) | 51/51 (100%) | 478/ 527 (90.7%) | 51/100 (51.0%) | 478/478 (100%) | 529/ 578 (91.5%) |
| | Female (N=651, 53.0%) | 76/76 (100%) | 499/575 (86.8%) | 76/152 (50.0%) | 499/499 (100%) | 575/ 651 (88.3%) |
| Age (-years old) | ≥ 65 (N=544, 44.3%) | 95/95 (100%) | 371/449 (82.6%) | 95/173 (54.9%) | 371/371 (100%) | 466/544 (85.7%) |
| | < 65 (N=685, 55.7%) | 32/32 (100%) | 606/653 (92.8%) | 32/79 (40.5%) | 606/606 (100%) | 638/685 (93.1%) |
| Etiology | Viral (N=880, 71.6%) | 100/100 (100%) | 690/780 (88.5%) | 100/190 (52.6%) | 690/690 (100%) | 790/880 (89.8%) |
| | Nonviral (N=349, 28.4%) | 27/27 (100%) | 287/322 (89.1%) | 27/62 (43.5%) | 287/287 (100%) | 314/349 (90.0%) |
| BMI ($kg/m^2$) | ≥ 23 (N=570, 46.4%) | 16/16 (100%) | 472/554 (85.2%) | 16/98 (16.3%) | 472/472 (100%) | 488/570 (85.6%) |
| | < 23 (N=659, 53.6%) | 111/111 (100%) | 505/548 (92.2%) | 111/154 (72.1%) | 505/505 (100%) | 616/659 (93.5%) |

PPV: positive predictive value, NPV: negative predictive value; BMI: body mass index

**Supplementary Table S2.** Diagnostic performance of the grip strength for the prediction of sarcoprenia (Data obtained from 2011 to 2015)

| Validation cohort | | Sensitivity | Specificity | PPV | NPV | Diagnostic accuracy |
|---|---|---|---|---|---|---|
| All cases from 2011 to 2015 (N=479, 100%) | | 67/67 (100%) | 353/412 (85.7%) | 67/126 (53.2%) | 353/353 (100%) | 420/479 (87.7%) |
| Gender | Male cases (N=230, 48.0%) | 28/28 (100%) | 180/202 (89.1%) | 28/50 (56.0%) | 180/180 (100%) | 208/ 230 (90.4%) |
| | Female cases (N=249, 52.0%) | 39/39 (100%) | 173/210 (82.4%) | 39/76 (51.3%) | 173/173 (100%) | 212/ 249 (85.1%) |
| Age (-years old) | ≥ 65 (N=219, 45.7%) | 44/44 (100%) | 141/175 (80.6%) | 44/78 (56.4%) | 141/141 (100%) | 185/219 (84.5%) |
| | < 65 (N=260, 54.3%) | 23/23 (100%) | 212/237 (89.5%) | 23/48 (47.9%) | 212/212 (100%) | 235/260 (90.4%) |
| Etiology | Viral (N=450, 93.9%) | 63/63 (100%) | 333/387 (86.0%) | 63/117 (53.8%) | 333/333 (100%) | 396/450 (88.0%) |
| | Nonviral (N=29, 6.1%) | 4/4 (100%) | 20/25 (80.0%) | 4/9 (44.4%) | 20/20 (100%) | 24/29 (82.8%) |
| BMI ($kg/m^2$) | ≥ 23 (N=162, 33.8%) | 6/6 (100%) | 123/156 (78.8%) | 6/39 (15.4%) | 123/123 (100%) | 129/162 (79.6%) |
| | < 23 (N=317, 66.2%) | 61/61 (100%) | 230/256 (89.8%) | 61/87 (70.1%) | 230/230 (100%) | 291/317 (91.8%) |

PPV: positive predictive value, NPV: negative predictive value; BMI: body mass index

**Supplementary Table S3.** Diagnostic performance of the grip strength for the prediction of sarcoprenia (Data obtained from 2016 to 2020)

| Validation cohort | | Sensitivity | Specificity | PPV | NPV | Diagnostic accuracy |
|---|---|---|---|---|---|---|
| All cases from 2016 to 2020 (N=750, 100%) | | 60/60(100%) | 624/690 (90.4%) | 60/126 (47.6%) | 624/624 (100%) | 684/750 (91.2%) |
| Gender | Male cases (N=348, 46.4%) | 23/23(100%) | 298/325 (91.7%) | 23/50 (46.0%) | 298/298 (100%) | 321/348 (92.2%) |
| | Female cases (N=402, 53.6%) | 37/37(100%) | 326/365 (89.3%) | 37/76 (48.7%) | 326/326(100%) | 363/402 (90.3%) |
| Age (-years old) | ≥ 65 (N=325, 43.3%) | 51/51 (100%) | 230/274 (83.9%) | 51/95 (53.7%) | 230/230 (100%) | 281/325 (86.5%) |
| | < 65 (N=425, 56.7%) | 9/9 (100%) | 394/416 (94.7%) | 9/31 (29.0%) | 394/394 (100%) | 403/425 (94.8%) |
| Etiology | Viral (N=430, 57.3%) | 37/37 (100%) | 357/393 (90.8%) | 37/73 (50.7%) | 357/357 (100%) | 394/430 (91.6%) |
| | Nonviral (N=320, 42.7%) | 23/23 (100%) | 267/297 (89.9%) | 23/53 (43.4%) | 267/267 (100%) | 290/320 (90.6%) |
| BMI | ≥ 23 (N=408, 54.4%) | 10/10 (100%) | 349/398 (87.7%) | 10/59 (16.9%) | 349/349 (100%) | 359/408 (88.0%) |
| | < 23 (N=342, 54.6%) | 50/50 (100%) | 275/292 (94.2%) | 50/67 (74.6%) | 275/275 (100%) | 325/342 (95.0%) |

PPV: positive predictive value, NPV: negative predictive value; BMI: body mass index
